# An Opening for Internes in the State Hospitals

**Published:** 1902-05

**Authors:** Frederick Peterson

**Affiliations:** President of the Commission in Lunacy; 4 West 50th Street, New York


### An Opening for Internes in the State Hospitals.

*Editor Buffalo Medical Journal :*

*Sir.*—Students about to graduate who are unable to secure positions in general hospitals, or young physicians whose terms are about to expire in general hospitals and who wish to enlarge their experience, are now offered an opportunity to enter the New York State Hospitals as internes or clinical assistants.

These positions provide lodging and board. Appointments are made for a year. Some twenty-eight positions will be opened in the fourteen state hospitals, situated in the following places in New York state: Utica, Buffalo, Gowanda (homeopathic), Binghamton, Kings Park, L.I.; Flatbush, Brooklyn; Central Islip, L.I.; Ward's Island, New York City (two hospitals), Rochester, Ogdensburg, Poughkeepsie, Willard, Middleton (homeopathic).

Although these are hospitals for the insane, yet they are so large that opportunities for experience in general medicine are abundant. Each hospital is equipped with clinico-pathological laboratory and apparatus, operating rooms, trained nurses, hydrotherapeutic and electrical devices and good medical libraries. The field for study in general medicine is excellent, and surgical operations of all kinds are frequently performed, either by resident or consulting surgeons. It is thought that many students who wish hospital experience and are unable to obtain it because of the relatively few places available in general hospitals, may be glad to learn that positions of this kind have been thrown open to them. It is believed that young physicians wishing hospital experience will profit by a year's residence in one of these hospitals, and such as desire to continue in special work would be eligible for appointments, subsequently, to salaried positions in the same service.

No examinations will be necessary, but application must be made in person with good references, directly to the medical superintendent of any of the above named hospitals or to the undersigned,

FREDERICK PETERSON, M. D.,
President of the Commission in Lunacy.

4 West 50th Street, New York, April 1, 1902.

